# Agreement of Benchmarking High Antimicrobial Usage Farms Based on Either Animal Treatment Index or Number of National Defined Daily Doses

**DOI:** 10.3389/fvets.2020.00638

**Published:** 2020-09-09

**Authors:** Dolf Kuemmerlen, Thomas Echtermann, Cedric Muentener, Xaver Sidler

**Affiliations:** ^1^Division of Swine Medicine, Department for Farm Animals, Vetsuisse Faculty, University of Zurich, Zurich, Switzerland; ^2^Institute of Veterinary Pharmacology and Toxicology, Vetsuisse Faculty, University of Zurich, Zurich, Switzerland

**Keywords:** antimicrobial usage, benchmarking, defined daily dose, animal treatment index, Suissano

## Abstract

**Introduction:** While treatment frequency as an indicator of antimicrobial consumption is often assessed using defined doses, it can also be calculated directly as an Animal Treatment Index (ATI). In this study, the correlation of calculating antimicrobial usage on Swiss pig farms using either national Defined Daily Doses (DDDch) or an ATI (number of treatments per animal per year) and the agreement between the different methods for the identification of high usage farms were investigated.

**Material and Methods:** The antimicrobial consumption of 893 Swiss pig herds was calculated separately for suckling piglets, weaned piglets, fattening pigs, lactating and gestating sows using the indicators nDDDch (number of DDDch) per animal per year and ATI. Correlations between the indicators were investigated by calculating Spearman's Rho coefficients. The 5, 10, and 25% highest usage farms were determined by applying both methods and the interrater reliability was described using Cohen's Kappa coefficients and visualized by Bland-Altman plots.

**Results:** The Spearman's Rho coefficients showed strong correlations (*r* > 0.5) between nDDDch/animal/year and ATI. The lowest coefficient was shown for the correlation of both indicators in gestating sows (*r* = 0.657) and the highest in weaned piglets (*r* = 0.910). Kappa coefficients identifying high usage farms were the highest in weaned piglets (*k* = 0.71, 0.85, and 0.91, respectively for 5, 10, and 25% most frequent users) and the lowest in gestating sows (*k* = 0.54, 0.58, and 0.55 for 5, 10, and 25% most frequent users).

**Conclusions:** In general, the investigated indicators showed strong correlations and a broad agreement in terms of the calculated levels of antimicrobial usage and the identification of high usage farms. Nevertheless, a certain proportion of the farms were defined differently depending on the indicator used. These differences varied by age category and were larger in all age categories except weaned piglets when a higher percentage benchmark was used to define high usage farms. These aspects should be considered when designing scientific studies or monitoring systems and considering which indicator to use.

## Introduction

Antimicrobial resistance poses a threat to both human and animal health (1). The use of antimicrobials is a key factor in the development of antimicrobial resistance in human and veterinary medicine (2, 3). Links between the use of antimicrobials and an increase or decrease in the frequency of antimicrobial resistance have already been described in many studies (4–10). Monitoring systems have been implemented in several countries as an important measure to investigate and control the use of antimicrobials on farms (11–15). In Switzerland, the Suissano Health Programme was developed and launched in 2015 to monitor antimicrobial usage on pig farms (16).

At the beginning of monitoring antimicrobial usage (AMU), only sales of antimicrobial products at the wholesale level were known, so analyses of antimicrobial usage could only be carried out based on these data. In order to be able to compare the calculated amounts of active substances between different populations of farm animals, the European Medicines Agency (EMA) introduced the so-called “Population Correction Unit (PCU).” For each animal species, an average value for the weight at the time of antibiotic treatment is assumed. In this way, all antibiotic quantities can be standardized with a value in kilograms of PCU and different animal species can be compared with each other. However, if the consumption of all antibiotics is given as a total quantity of active substance, no account is taken of the fact that the different classes of active substance, for example penicillins and fluoroquinolones, are used in different dosages (17). For this reason, other indicators were subsequently developed to describe the usage of antimicrobials (18, 19). In human medicine, Defined Daily Doses (DDD) were developed to analyze the usage of various medicines. In analogy, DDD have also been published for veterinary medicine (20). These units describe the daily amount of active ingredient required for the treatment of an animal with a standardized weight. Recently, the EMA published guidelines for the development and publication of indicators for the description of antimicrobial use in veterinary medicine, but has also made such indicators available in the form of Defined Daily Doses (DDDvet) and Defined Course Doses (DCDvet) (21). The DDDvet values based on data from nine European countries have been shown in some cases to differ considerably from the dosages specified in the respective national summaries of product characteristics (SPCs). Therefore, national DDDs and DCDs have been developed in several countries (15, 22, 23). For this reason, separate Defined Daily Doses (DDDch) and Defined Course Doses (DCDch) were developed for Switzerland, some of which consequently showed considerable deviations from the indicators published by the EMA (21, 23–26). Due to these discrepancies, some authors consider the so-called treatment incidence based on used daily doses, which describes the proportion of treated animals at a specific point in time as the method of choice to describe the usage of antimicrobials (19, 27–30). Moreover, an Animal Treatment Index (ATI) was introduced, which described the proportion of treated animals on a farm (31).

The indicators described above are not only used in various monitoring systems to describe the usage of antimicrobials, but in most cases can also be used to identify high usage farms by setting a benchmark, and to determine interventions to lower AMU (11, 32). In this way a successful reduction of antimicrobial usage in pig farms could be demonstrated (32, 33). As the calculation methods sometimes varied considerably, differences in the identification of high usage farms were also possible: a recent study by Kasabova et al. (33) presented such differences for the evaluation of antimicrobial usage in pig and poultry farms in Germany based on Defined Daily Doses or the Used Daily Doses (17, 34, 35).

The joint Suissano/Safety+ Health Programme was launched in 2018 in cooperation of Swiss pig producers, veterinary authorities, pig trading companies and retailers. The aim of the programme was to improve transparency concerning AMU. For participating farms, it is obligatory to record each antimicrobial treatment of pigs by an electronic treatment journal, which is run on personal computers or smart phone applications. All treatments are allocated to five categories (suckling piglets, weaned piglets, fattening pigs, lactating and gestating sows). Each participating farm is quarterly provided with a feedback concerning AMU including a comparison with the AMU levels of all other participating farms. Although being a voluntary programme, the number of participating farms increases every year and by the end of 2020 it is expected that around 2200 farms, or over 50% of all Swiss pig herds will be part of the programme (Service and competence center of the Swiss pig industry, SUISAG; personal communication).

In the present study, antimicrobial usage in five different age categories (suckling piglets, weaned piglets, fattening pigs, gestating and lactating sows) was calculated for 893 pig herds participating in the Suissano/Safety+ Health Programme, either as an ATI or number of DDDch (nDDDch) per animal per year. Correlations between the two indicators and differences in the definition of high usage farms were investigated and visualized.

## Materials and Methods

### Data Collection

All farms involved in the study participated in the Suissano/Safety+ Health Programme in Switzerland. Farmers recorded their antimicrobial usage using electronic treatment journals which were linked to a central database from which all data used in this study were retrieved. For each individual treatment, in addition to the antimicrobial product used and the amount administered, the number of animals treated and the duration of the treatment were recorded. Each treatment was assigned to an age category (suckling piglets, weaned piglets, fattening pigs, gestating sows, and lactating sows). In addition, the numbers of suckling piglets, weaned piglets and fattening pigs produced from all the herds participating in this study once a year had to be reported by the farmer and were registered in the electronic treatment journal, as well as the number of gestating and lactating sows housed on the study farms.

A total of 893 farms provided data for our study. Three hundred and ninety-nine were fattening farms providing data concerning fattening pigs and 481 were breeding farms (housing sows, suckling piglets, and weaned piglets), of which 190 were connected to a sow pool system (housing lactating sows, suckling piglets and weaned piglets or only gestating sows). Thirteen farms kept both weaned piglets and fattening pigs. Two hundred and seventy-seven of the 481 breeding farms also kept at least 30 fattening pigs (farrow-finish farms) and thus provided data concerning all age categories (Table 1).

**Table 1:** Number of farms out of the 893 study farms providing complete datasets of AMU for each age category; minimum (min), median, maximum (max), and the total number of animals of the respective age category on these farms.

	**Number of farms**	**Total number of animals**	**Animals housed or produced**		
			**Median**	**Min**	**Max**
Lactating sows	462	12,176	22	5	140
Gestating sows	319	30,522	75	14	536
Suckling piglets	404	1,081,410	2,200	180	16,500
Weaned piglets	360	878,154	2,000	100	15,000
Fattening pigs	531	713,661	1,050	30	8,000

For each age category, data were only included in the dataset if they had been recorded continuously for the study period. The second inclusion criteria meant that data must be entered in the electronic treatment journal no later than 7 days after application, according to the requirements of the Suissano/Safety+ Health Programme.

The total amount of each active substance, administered during the study period (1 year) was added up and divided by the corresponding DDDch value defined by Echtermann et al. (24) multiplied by the standard weight of the corresponding animal group (suckling piglets: 4 kg; weaned piglets: 12 kg; fattening pigs: 50 kg; sows: 220 kg) (36). This calculation was performed separately for each active substance used and then the calculated nDDDch of all active substances used in each age category were summed up. The results for nDDDch were divided by the number of animals kept (sows) or produced (suckling piglets, weaned piglets, fattening pigs) during the study period (1. October 2018 to 30. September 2019). The nDDDch/animal/year was calculated according to the following formula.

$$\begin{array}{l} \;\frac{\sum amount\;of\;active\;substance\;used\;per\;year\;\left(mg\right)}{DDDch\;\left(\frac{mg}{kg}\right)*\;SW\;\left(kg\right)*\;number\;of\;animals\;housed\;per\;year}\\ =nDDDch/animal/year\; \end{array}$$

The ATI was calculated for each active substance and age category by adding up all treatments performed (number of treatments, NT) and dividing the sum by the number of animals kept or produced during the study period. One treatment was equivalent to one application per animal per day. If, for example, 20 animals were treated on 3 days during a therapy, this corresponded to 60 treatments. For treatments with products containing several antimicrobial agents, each agent was individually evaluated as a treatment.

For treatments with long-acting products, multiplying factors corresponding to the duration of the pharmaceutical activity were used to adjust the treatment duration. However, since these factors only served to compare the usage of antimicrobial ingredients with different pharmaceutical activity, it had no influence on the comparison of the two calculation methods and was disregarded in this study. Recorded treatments that could be clearly identified as incorrect, due to markedly differing numbers of animals, treatment duration or quantities of Veterinary Medical Products (VMPs), were removed from the analysis.

### Statistical Analyses

All datasets were prepared with Microsoft Excel® Version 16.30. Statistical analysis was carried out using IBM SPSS® Version 25. All datasets were tested for normal distribution by Shapiro-Wilk test. For not normally distributed datasets, correlations between the indicators were investigated by calculating Spearman's Rho coefficients. Further, the relationship between both indicators was visualized by scatterplots and by linear regression lines using a generalized linear model for the age categories with the lowest and the highest Spearman's Rho coefficients. The interrater reliability between the tested calculation methods for identifying the 5, 10, and 25% highest usage farms was described using Cohen's Kappa coefficients and visualized by Bland-Altman plots of means and differences between ATI and nDDDch/animal/year. Values of ±1.96 multiplied with the standard deviation of the differences mentioned were defined as outliers. The percentage of farms for which agreement could be found when determining frequent users by either nDDDch/animal/year or ATI was calculated for each age category.

## Results

Tables 2–6 give an overview of the antimicrobial classes used in the study farms in the different age categories. A total AMU of 649,290 treatments, respectively, 557,830 DDDch was calculated for the study period. This represents a deviation of 14%, when calculating NT or nDDDch, respectively. For the different classes of active substances, strongly deviating agreement of the indicators could be observed: While the results for the usage of sulfonamides in lactating sows were almost identical when calculated with both indicators, the NT with tetracyclines in weaning piglets was almost three times higher than the nDDDch and on the other hand, the nDDDch for treatments with aminoglycosides was 2.5 times higher than the NT. Table 7 shows the correlation between the calculations of the AMU as ATI or nDDDch/animal/year and the agreement of the definition of 5, 10, and 25% high usage farms, depending on the method chosen. The highest correlation coefficients between both indicators could be found for lactating sows and weaned piglets and the lowest for gestating sows and fattening pigs. Both agreement (a) and the interrater reliability (Kappa coefficient) of the investigated indicators were higher when identifying the 5 and 10% high usage farms, compared to the identified 25% high usage farms (Table 7). Exception were the weaned piglets, where Kappa coefficient became higher when larger proportions of farms were identified as high users. Generally, weaned piglets showed the best agreement (≥96%) irrespective of the percentage of high usage farms identified and the best correlation between the indicators.

**Table 2:** Total, minimum (min), median, maximum (max), 10% (0.1), 25% (0.25), 75% (0.75), 90% (0.9) percentiles and standard deviation (SD) of AMU in suckling piglets of the study farms measured using the indicators NT, ATI, nDDDch, and nDDDch/animal/year (nDDDch/a/y) displayed by active substance (AS).

	**Suckling piglets**									
**AS**	**Indicator**	**Total**	**Min**	**0.10**	**0.25**	**Median**	**0.75**	**0.90**	**Max**	**SD**
Polypeptides	NT	11,095	0.00	0.00	0.00	0.00	0.00	52	2,184	140
	ATI	0.06	0.00	0.00	0.00	0.00	0.00	0.02	0.26	0.03
	nDDDch	6,508	0.00	0.00	0.00	0.00	0.00	29	1,051	82
	nDDDch/a/y	0.01	0.00	0.00	0.00	0.00	0.00	0.01	0.39	0.03
Cephalosporines	NT	38	0.00	0.00	0.00	0.00	0.00	0.00	38	1.89
	ATI	0.00	0.00	0.00	0.00	0.00	0.00	0.00	0.03	0.00
	nDDDch	15	0.00	0.00	0.00	0.00	0.00	0.00	15	0.75
	nDDDch/a/y	0.00	0.00	0.00	0.00	0.00	0.00	0.00	0.01	0.00
Penicillins	NT	85,868	0.00	0.00	6	36	150	506	5,429	524
	ATI	0.08	0.00	0.00	0.00	0.00	0.00	0.00	0.00	0.00
	nDDDch	69,295	0.00	0.00	1.12	19	144	488	5,263	467
	nDDDch/a/y	0.06	0.00	0.00	0.00	0.00	0.00	0.00	0.00	0.00
Fluoroquinolones	NT	6,453	0.00	0.00	0.00	0.00	0.00	37	678	61
	ATI	0.01	0.00	0.00	0.00	0.00	0.00	0.02	0.31	0.02
	nDDDch	9,600	0.00	0.00	0.00	0.00	0.00	29	1,520	123
	nDDDch/a/y	0.01	0.00	0.00	0.00	0.00	0.00	0.01	0.65	0.05
Aminoglycosides	NT	13,524	0.00	0.00	0.00	0.00	1.00	62	1,574	142
	ATI	0.01	0.00	0.00	0.00	0.00	0.00	0.03	0.33	0.03
	nDDDch	31,598	0.00	0.00	0.00	0.00	0.72	148	3,921	343
	nDDDch/a/y	0.03	0.00	0.00	0.00	0.00	0.00	0.05	1.19	0.09
Macrolides	NT	243	0.00	0.00	0.00	0.00	0.00	0.00	120	7.09
	ATI	0.00	0.00	0.00	0.00	0.00	0.00	0.00	0.17	0.01
	nDDDch	244	0.00	0.00	0.00	0.00	0.00	0.00	104	7.31
	nDDDch/a/y	0.00	0.00	0.00	0.00	0.00	0.00	0.00	0.13	0.01
Tetracyclines	NT	11,973	0.00	0.00	0.00	0.00	0.00	60	1,760	144
	ATI	0.01	0.00	0.00	0.00	0.00	0.00	0.03	0.54	0.05
	nDDDch	8,332	0.00	0.00	0.00	0.00	0.00	39	1,329	98
	nDDDch/a/y	0.01	0.00	0.00	0.00	0.00	0.00	0.02	0.47	0.04
Trimethoprim	NT	4,202	0.00	0.00	0.00	0.00	0.00	16	490	45
	ATI	0.00	0.00	0.00	0.00	0.00	0.00	0.01	0.22	0.02
	nDDDch	6,241	0.00	0.00	0.00	0.00	0.00	15	884	76
	nDDDch/a/y	0.01	0.00	0.00	0.00	0.00	0.00	0.01	0.24	0.03
Sulfonamides	NT	4,445	0.00	0.00	0.00	0.00	0.00	20	490	45
	ATI	0.00411	0.00	0.00	0.00	0.00	0.00	0.01	0.22	0.02
	nDDDch	6,483	0.00	0.00	0.00	0.00	0.00	18	884	76
	nDDDch/a/y	0.01	0.00	0.00	0.00	0.00	0.00	0.01	0.24	0.03
Total	NT	137,841	1.00	24	45	144	352	938	5,934	677
	ATI	0.13	0.00	0.01	0.02	0.07	0.15	0.27	1.50	0.15
	nDDDch	138,316	0.24	20	32	125	378	887	6,042	722
	nDDDch/a/y	0.13	0.00	0.01	0.02	0.06	0.16	0.35	1.83	0.19

**Table 3:** Total, minimum (min), median, maximum (max), 10% (0.1), 25% (0.25), 75% (0.75), 90% (0.9) percentiles and standard deviation (SD) of AMU in weaned piglets of the study farms measured using the indicators NT, ATI, nDDDch, and nDDDch/animal/year (nDDDch/a/y) displayed by active substance (AS).

	**Weaned piglets**									
**AS**	**Indicator**	**Total**	**Min**	**0.10**	**0.25**	**Median**	**0.75**	**0.90**	**Max**	**SD**
Polypeptides	NT	116,729	1.00	102	270.	740.	1,800	3,060	21,340	2,986
	ATI	0.13	0.00	0.00	0.00	0.00	0.00	0.40	2.40	0.38
	nDDDch	102,472	0.10	85	192	668	1,653	2,974	14,503	2,412
	nDDDch/a/y	0.12	0.00	0.00	0.00	0.00	0.00	0.31		0.35
Cephalosporines	NT	3	3.00	3.00	3.00	3.00	3.00	3.00	3.00	–
	ATI	0.00	0.00	0.00	0.00	0.00	0.00	0.00	0.00	0.00
	nDDDch	6.25	6.25	6.25	6.25	6.25	6.25	6.25	6.25	–
	nDDDch/a/y	0.00	0.00	0.00	0.00	0.00	0.00	0.00	0.00	0.00
Penicillins	NT	51,621	0.00	0.00	0.00	9.00	40	205	10,619	717
	ATI	0.06	0.00	0.00	0.00	0.00	0.02	0.07	1.76	0.17
	nDDDch	52,086	0.00	0.00	0.00	3.33	40	196	10,700	718
	nDDDch/a/y	0.06	0.00	0.00	0.00	0.00	0.02	0.08	1.89	0.17
Fluoroquinolones	NT	2,487	1.00	2.00	4.00	12	39	253	717	157
	ATI	0.00	0.00	0.00	0.00	0.00	0.00	0.00	0.42	0.02
	nDDDch	3,626	0.42	2.11	5.00	13	33	236	1,296	268
	nDDDch/a/y	0.00	0.00	0.00	0.00	0.00	0.00	0.00	0.42	0.03
Aminoglycosides	NT	2,550	1.00	3.00	4.75	11	46	81	37	6
	ATI	0.00	0.00	0.00	0.00	0.00	0.00	0.01	0.11	0.01
	nDDDch	4,250	1.25	2.72	6.67	22	77	152	534	94
	nDDDch/a/y	0.00	0.00	0.00	0.00	0.00	0.00	0.01	0.11	0.01
Macrolides	NT	28,938	10	38	85	231	460	872	12,030	1,770
	ATI	0.03	0.00	0.00	0.00	0.00	0.00	0.03	2.51	0.18
	nDDDch	24,993	23	46	11	233	497	815	8,495	1,244
	nDDDch/a/y	0.03	0.00	0.00	0.00	0.00	0.00	0.04	1.77	0.15
Tetracyclines	NT	98,860	1.00	5.00	18	71	429	1,480	21,340	2,914
	ATI	0.11	0.00	0.00	0.00	0.00	0.01	0.16	2.96	0.31
	nDDDch	40,316	0.20	4.64	11	45	320	798	8,495	928
	nDDDch/a/y	0.05	0.00	0.00	0.00	0.00	0.00	0.09	1.77	0.18
Trimethoprim	NT	11,313	1.00	2.30	8.75	30	139	359	3,652	486
	ATI	0.01	0.00	0.00	0.00	0.00	0.00	0.01	0.73	0.06
	nDDDch	23,306	1.17	3.31	8.00	332	161	371	3,347	465
	nDDDch/a/y	0.03	0.00	0.00	0.00	0.00	0.00	0.01	1.19	0.09
Sulfonnamides	NT	46,981	0.00	0.00	0.00	0.00	5.75	211	12,030	789
	ATI	0.05	0.00	0.00	0.00	0.00	0.00	0.11	2.51	0.21
	nDDDch	65,870	0.00	0.00	0.00	0.00	6.84	206	8,084	605
	nDDDch/a/y	0.08	0.00	0.00	0.00	0.00	0.00	0.11	1.96	0.21
Pleuromutilins	NT	8,104	6.00	164	400	1,269	1,671	2,869	3,667	1,429
	ATI	0.01	0.00	0.00	0.00	0.00	0.00	0.00	0.76	0.06
	nDDDch	7,014	4.00	93	225	879.00	2,136	3,770	4,860	1,991
	nDDDch/a/y	0.01	0.00	0.00	0.00	0.00	0.00	0.00	1.01	0.06
Total	NT	367,586	1.00	4.00	20	98.00	590	2,135	42,842	3,741
	ATI	0.31	0.00	0.00	0.01	0.04	0.29	1.12	7.52	0.81
	nDDDch	300,168	0.10	3.58	15	91	642	1,933	25,076	2,209
	nDDDch/a/y	0.34	0.00	0.00	0.01	0.06	0.27	0.97	17	0.67

**Table 4:** Total, minimum (min), median, maximum (max), 10% (0.1), 25% (0.25), 75% (0.75), 90% (0.9) percentiles and standard deviation (SD) of AMU in fattening pigs of the study farms measured using the indicators NT, ATI, nDDDch and nDDDch/animal/year (nDDDch/a/y) displayed by active substance (AS).

	**Fattening pigs**									
**AS**	**Indicator**	**Total**	**Min**	**0.10**	**0.25**	**Median**	**0.75**	**0.90**	**Max**	**SD**
Cephalosporines	NT	9	2.00	2.20	2.50	3.00	3.50	3.80	4.00	1.00
	ATI	0.00	0.00	0.00	0.00	0.00	0.00	0.00	0.01	0.00
	nDDDch	16	3.75	4.00	4.38	5.00	6.00	6.60	7.00	1.64
	nDDDch/a/y	0.00	0.00	0.00	0.00	0.00	0.00	0.00	0.01	0.00
Penicillins	NT	45,976	0.00	2.00	5.00	16	48	134	7,622	416
	ATI	0.06	0.00	0.00	0.01	0.02	0.05	0.10	2.72	0.22
	nDDDch	39,141	0.00	0.08	0.98	7.20	40	120	7,965	425
	nDDDch/a/y	0.05	0.00	0.00	0.00	0.01	0.04	0.11	3.19	0.24
Fluoroquinolone	NT	144	1.00	1.20	3.00	3.50	17	36	44	15
	ATI	0.00	0.00	0.00	0.00	0.00	0.00	0.00	0.07	0.00
	nDDDch	160	0.70	1.92	3.15	7.00	21	30	39	13
	nDDDch/a/y	0.00	0.00	0.00	0.00	0.00	0.00	0.00	0.08	0.01
Aminoglycosides	NT	1,524	1.00	1.80	3.00	6.00	18	42	96	20
	ATI	0.00	0.00	0.00	0.00	0.00	0.00	0.01	0.14	0.01
	nDDDch	1,919	0.56	1.56	3.00	8.70	22	53	223	31
	nDDDch/a/y	0.00	0.00	0.00	0.00	0.00	0.00	0.01	0.15	0.01
Macrolides	NT	11,919	3.00	3.00	7.00	95	811	1,685	7,000	1,859
	ATI	0.02	0.00	0.00	0.00	0.00	0.00	0.00	2.80	0.16
	nDDDch	12,296	0.20	1.23	7.05	73	449	1,680	8,330	2,219
	nDDDch/a/y	0.02	0.00	0.00	0.00	0.00	0.00	0.00	3.33	0.16
Tetracyclines	NT	25,152	1.00	2.00	4.00	10.00	37	224	7,000	879
	ATI	0.04	0.00	0.00	0.00	0.00	0.00	0.01	2.80	0.21
	nDDDch	20,633	0.20	1.20	2.42	8.90	18	94	8,330	910
	nDDDch/a/y	0.03	0.00	0.00	0.00	0.00	0.00	0.01	3.33	0.19
Trimethoprim	NT	8,966	1.00	1.70	2.75	6.00	183	493	6,000	1,133
	ATI	0.01	0.00	0.00	0.00	0.00	0.00	0.00	4.00	0.23
	nDDDch	5,308	0.70	1.74	3.08	6.13	106	363	3,199	606
	nDDDch/a/y	0.01	0.00	0.00	0.00	0.00	0.00	0.00	2.13	0.13
Sulfonamides	NT	29,324	0.00	0.00	0.00	0.00	0.00	0.00	12,000	622.20
	ATI	0.04	0.00	0.00	0.00	0.00	0.00	0.00	8.00	0.49
	nDDDch	22,343	0.00	0.00	0.00	0.00	0.00	0.00	8,330	473
	nDDDch/a/y	0.03	0.00	0.00	0.00	0.00	0.00	0.00		0.31
Pleuromutilines	NT	2,264	1.44	3.02	4.76	15	38	388	1,333	368
	ATI	0.00	0.00	0.00	0.00	0.00	0.00	0.00	0.35	0.02
	nDDDch	2,068	2.00	3.00	6.25	18	62	723	1,000	356
	nDDDch/a/y	0.00	0.00	0.00	0.00	0.00	0.00	0.00	0.65	0.03
Total	NT	125,278	1.00	3.00	8.00	24	75	236	25,000	1,455
	ATI	0.18	0.00	0.00	0.01	0.03	0.06	0.15	12.05	0.91
	nDDDch	103,884	0.05	1.20	3.62	16	62	178	32,955	1,558
	nDDDch/a/y	0.15	0.00	0.00	0.00	0.02	0.05	0.15	13	0.75

**Table 5:** Total, minimum (min), median, maximum (max), 10% (0.1), 25% (0.25), 75% (0.75), 90% (0.9) percentiles and standard deviation (SD) of AMU in lactating sows of the study farms measured using the indicators NT, ATI, nDDDch and nDDDch/animal/year (nDDDch/a/y) displayed by active substance (AS).

	**Lactating sows**									
**AS**	**Indicator**	**Total**	**Min**	**0.10**	**0.25**	**Median**	**0.75**	**0.90**	**Max**	**SD**
Cephalosporines	NT	21	0.00	0.00	0.00	0.00	0.00	0.00	10	0.63
	ATI	0.00	0.00	0.00	0.00	0.00	0.00	0.00	0.50	0.03
	nDDDch	17	0.00	0.00	0.00	0.00	0.00	0.00	7.91	0.50
	nDDDch/a/y	0.00	0.00	0.00	0.00	0.00	0.00	0.00	0.40	0.03
Penicillins	NT	3,212	0.00	0.00	0.00	2.00	8.00	15	244	17
	ATI	0.26	0.00	0.00	0.00	0.09	0.30	0.66	6.35	0.56
	nDDDch	2,528	0.00	0.00	0.00	0.28	4.55	12	295	19
	nDDDch/a/y	0.21	0.00	0.00	0.00	0.01	0.18	0.50	6.85	0.63
Fluoroquinolone	NT	271	0.00	0.00	0.00	0.00	0.00	1.00	31	2.44
	ATI	0.02	0.00	0.00	0.00	0.00	0.00	0.05	0.97	0.10
	nDDDch	291	0.00	0.00	0.00	0.00	0.00	1.61	32	2.63
	nDDDch/a/y	0.02	0.00	0.00	0.00	0.00	0.00	0.06	1.02	0.11
Aminoglycosides	NT	1,587	0.00	0.00	0.00	0.00	2.00	11	204	12
	ATI	0.13	0.00	0.00	0.00	0.00	0.10	0.41	10	0.55
	nDDDch	1,573	0.00	0.00	0.00	0.00	2.27	10	189	11
	nDDDch/a/y	0.13	0.00	0.00	0.00	0.00	0.10	0.42	9.46	0.53
Macrolides	NT	8	0.00	0.00	0.00	0.00	0.00	0.00	7.00	0.33
	ATI	0.00	0.00	0.00	0.00	0.00	0.00	0.00	0.26	0.01
	nDDDch	12	0.00	0.00	0.00	0.00	0.00	0.00	12	0.55
	nDDDch/a/y	0.00	0.00	0.00	0.00	0.00	0.00	0.00	0.44	0.02
Tetracyclines	NT	347	0.00	0.00	0.00	0.00	0.00	0.00	88	5.26
	ATI	0.03	0.00	0.00	0.00	0.00	0.00	0.00	1.83	0.15
	nDDDch	244	0.00	0.00	0.00	0.00	0.00	0.00	80	4.31
	nDDDch/a/y	0.02	0.00	0.00	0.00	0.00	0.00	0.00	1.67	0.12
Trimethoprim	NT	2,251	0.00	0.00	0.00	2.00	6.00	14.00	92.00	8.31
	ATI	0.18	0.00	0.00	0.00	0.08	0.29	0.56	2.92	0.32
	nDDDch	2,222	0.00	0.00	0.00	1.61	6.75	13.96	64.36	7.91
	nDDDch/a/y	0.18	0.00	0.00	0.00	0.08	0.29	0.51	2.65	0.34
Sulfonamides	NT	2,262	0.00	0.00	0.00	2.00	6.00	14.00	92.00	8.31
	ATI	0.19	0.00	0.00	0.00	0.08	0.29	0.56	2.92	0.32
	nDDDch	2,237	0.00	0.00	0.00	1.64	6.80	13.96	64.36	7.92
	nDDDch/a/y	0.18	0.00	0.00	0.00	0.08	0.30	0.51	2.65	0.34
Total	NT	9,959	1.00	3.00	6.00	13	25	44	331	32
	ATI	0.82	0.04	0.15	0.29	0.59	1.00	1.85	16.55	1.13
	nDDDch	9,124.00	0.05	2.27	4.78	11	25	41.73	390	30
	nDDDch/a/y	0.75	0.00	0.11	0.23	0.53	0.98	1.77	15	1.14

**Table 6:** Total, minimum (min), median, maximum (max), 10% (0.1), 25% (0.25), 75% (0.75), 90% (0.9) percentiles and standard deviation (SD) of AMU in gestating sows of the study farms measured using the indicators NT, ATI, nDDDch and nDDDch/animal/year (nDDDch/a/y) displayed by active substance (AS).

	**Gestating sows**									
**AS**	**Indicator**	**Total**	**Min**	**0.10**	**0.25**	**Median**	**0.75**	**0.90**	**Max**	**SD**
Cephalosporines	NT	74	0.00	0.00	0.00	0.00	0.00	0.00	30	2.13
	ATI	0.00	0.00	0.00	0.00	0.00	0.00	0.00	0.21	0.02
	nDDDch	76	0.00	0.00	0.00	0.00	0.00	0.00	0.00	0.00
	nDDDch/a/y	0.00	0.00	0.00	0.00	0.00	0.00	0.00	0.00	0.00
Penicillins	NT	6,908	0.00	1.00	4.00	9.00	20	46	540	47
	ATI	0.23	0.00	0.02	0.05	0.11	0.28	0.50	6.02	0.50
	nDDDch	4,438	0.00	0.07	0.57	3.55	12	30	650	46
	nDDDch/a/y	0.15	0.00	0.00	0.01	0.05	0.15	0.33	6.50	0.47
Fluoroquinolones	NT	55	0.00	0.00	0.00	0.00	0.00	0.00	16	1.31
	ATI	0.00	0.00	0.00	0.00	0.00	0.00	0.00	0.27	0.02
	nDDDch	68	0.00	0.00	0.00	0.00	0.00	0.00	26	1.79
	nDDDch/a/y	0.00	0.00	0.00	0.00	0.00	0.00	0.00	0.44	0.03
Aminoglycosides	NT	724	0.00	0.00	0.00	0.00	0.00	5.00	88	8.38
	ATI	0.02	0.00	0.00	0.00	0.00	0.00	0.09	0.73	0.08
	nDDDch	634	0.00	0.00	0.00	0.00	0.00	4.53	91	7.60
	nDDDch/a/y	0.02	0.00	0.00	0.00	0.00	0.00	0.07	0.58	0.07
Macrolides	NT	29	0.00	0.00	0.00	0.00	0.00	0.00	18	1.08
	ATI	0.00	0.00	0.00	0.00	0.00	0.00	0.00	0.13	0.01
	nDDDch	32	0.00	0.00	0.00	0.00	0.00	0.00	25	1.41
	nDDDch/a/y	0.00	0.00	0.00	0.00	0.00	0.00	0.00	0.18	0.01
Tetracyclines	NT	381	0.00	0.00	0.00	0.00	0.00	0.20	93	6.62
	ATI	0.01	0.00	0.00	0.00	0.00	0.00	0.00	0.66	0.07
	nDDDch	155	0.00	0.00	0.00	0.00	0.00	0.00	27	2.62
	nDDDch/a/y	0.01	0.00	0.00	0.00	0.00	0.00	0.00	0.25	0.03
Trimethoprim	NT	221	0.00	0.00	0.00	0.00	0.00	1.00	25	2.74
	ATI	0.01	0.00	0.00	0.00	0.00	0.00	0.01	0.34	0.03
	nDDDch	228	0.00	0.00	0.00	0.00	0.00	1.39	29	2.97
	nDDDch/a/y	0.01	0.00	0.00	0.00	0.00	0.00	0.01	0.39	0.04
Sulfonamides	NT	234	0.00	0.00	0.00	0.00	0.00	1.20	25	2.90
	ATI	0.01	0.00	0.00	0.00	0.00	0.00	0.02	0.34	0.03
	nDDDch	243	0.00	0.00	0.00	0.00	0.00	0.00	0.00	0.00
	nDDDch/a/y	0.01	0.00	0.00	0.00	0.00	0.00	0.00	0.00	0.00
Total	NT	8,626	1.00	3.00	6.00	12.00	27	65	548	51
	ATI	0.28	0.01	0.04	0.08	0.17	0.37	0.64	6.32	0.54
	nDDDch	5,873	0.02	0.87	2.73	6.67	18	340	660	49
	nDDDch/a/y	0.19	0.00	0.01	0.03	0.10	0.23	0.49	6.60	0.51

**Table 7:** Agreement (*a*) in percent when defining 5, 10, and 25% high usage farms by calculating AMU with either ATI or nDDDch/animal/year for five age categories (fattening pigs, weaned piglets, suckling piglets, lactating and gestating sows), interrater reliability between both indicators expressed as Kappa coefficient (*k*) and correlation between both indicators displayed using Spearman's Rho (*r*) coefficients.

	**5% High usage**		**10% High usage**		**25% High usage**		**Correlation**
	**a**	**k**	**a**	**k**	**a**	**k**	**r**
Fattening pigs	98%	0.844	93%	0.671	75%	0.510	0.673
Weaned piglets	97%	0.708	97%	0.846	96%	0.911	0.910
Suckling piglets	96%	0.643	91%	0.528	77%	0.554	0.793
Lactating sows	96%	0.634	96%	0.771	88%	0.77	0.889
Gestating sows	95%	0.539	92%	0.583	77%	0.553	0.657

**Figure 1:**
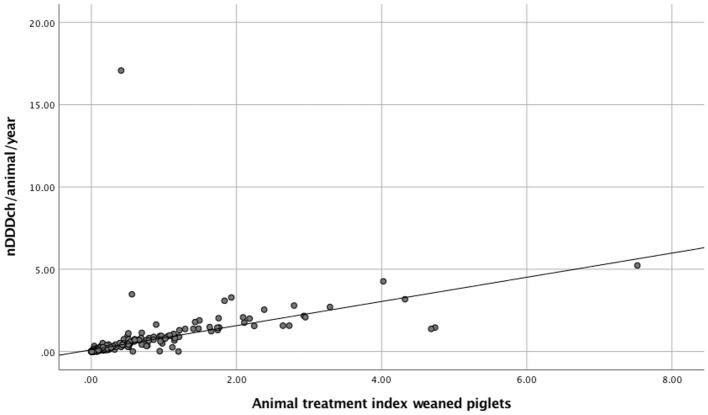
Scatterplots and linear regression of AMU calculated either as Animal Treatment Index (ATI) or the number of DDDch/animal/year (nDDDch/animal/year) for weaned piglets from 360 study farms.

**Figure 2:**
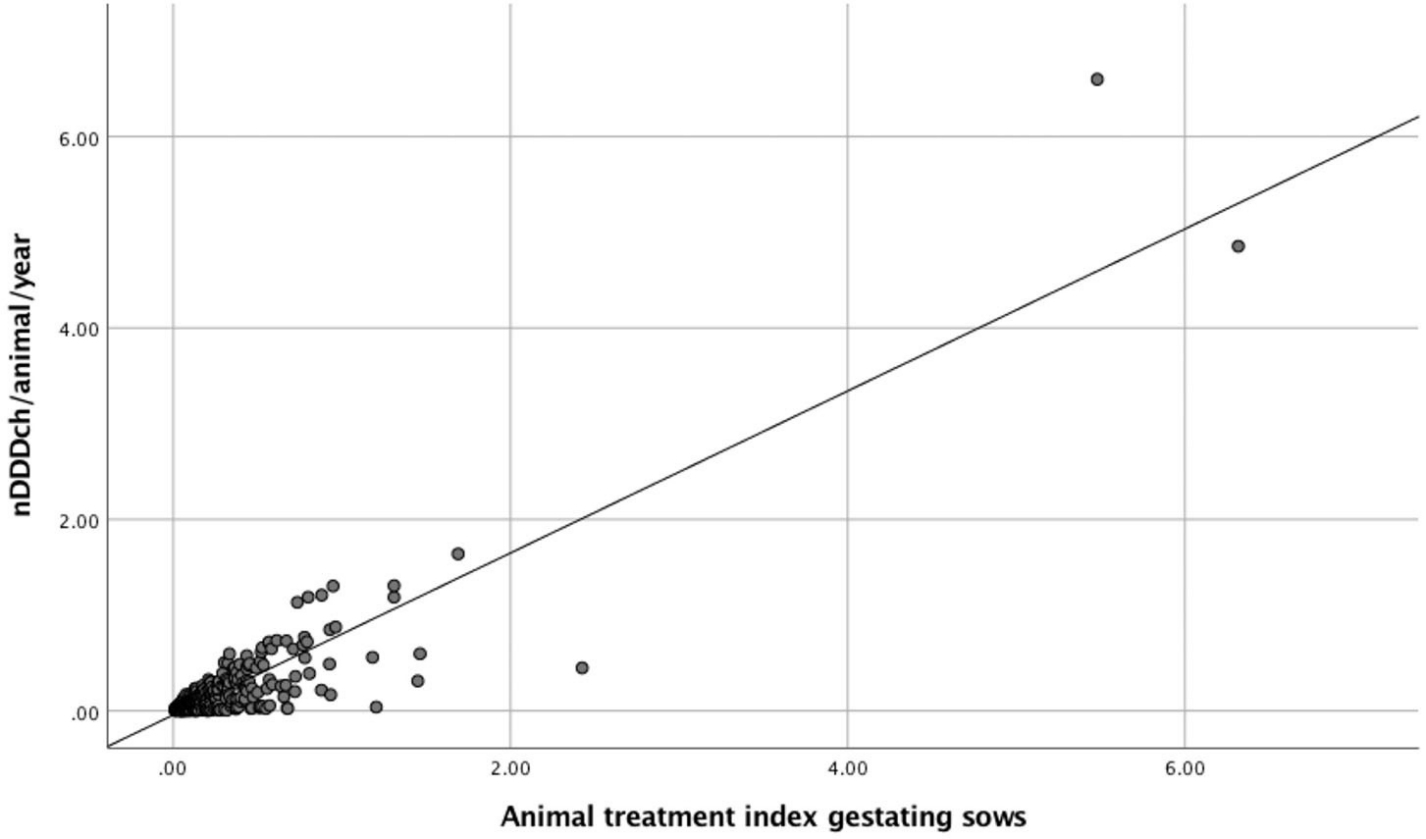
Scatterplots and linear regression of AMU calculated either as Animal Treatment Index (ATI) or the number of DDDch/animal/year (nDDDch/animal/year) for gestating sows from 319 study farms.

**Figure 3:**
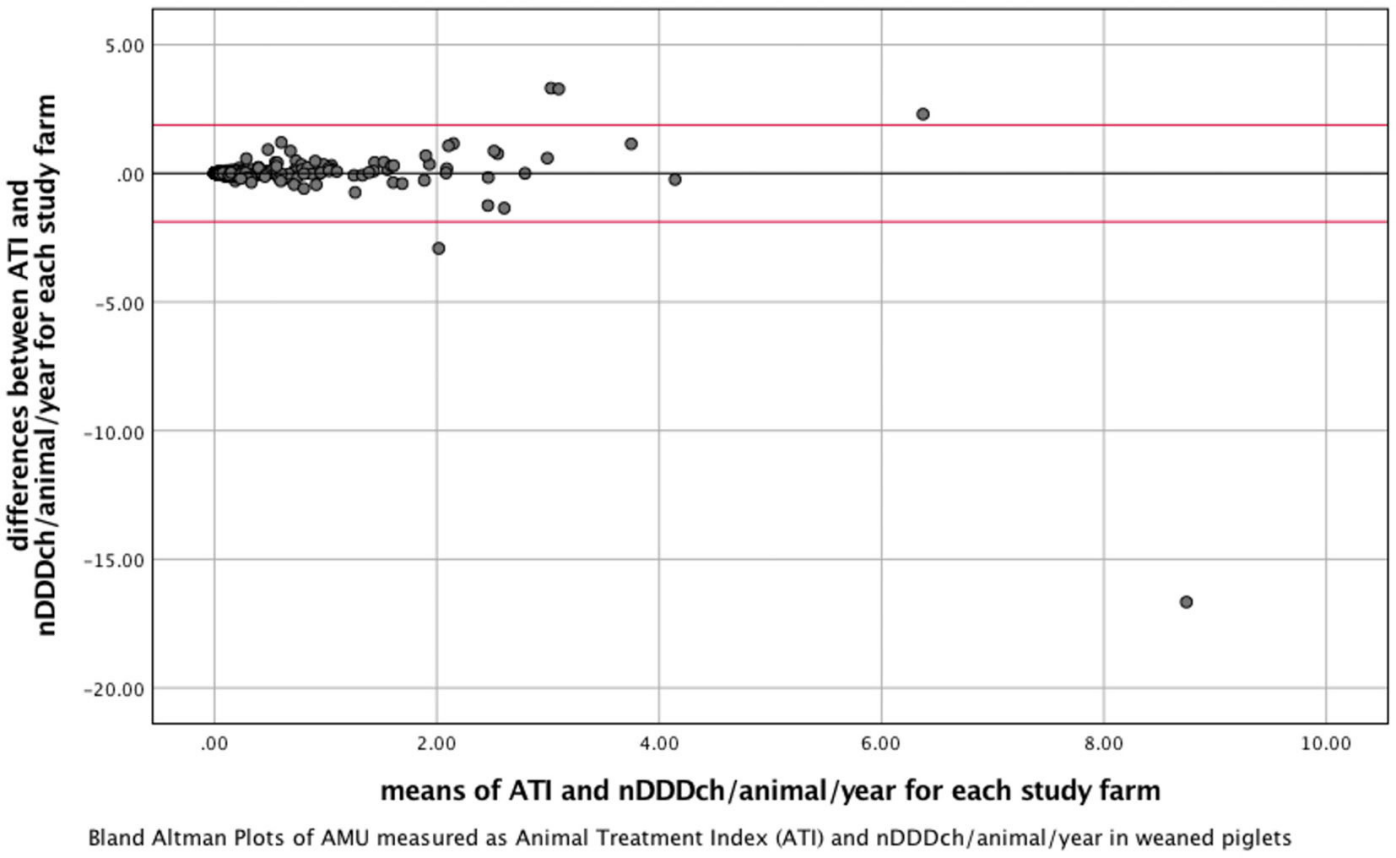
Bland-Altman Plots visualizing interrater reliability of the indicators Animal Treatment Index (ATI) and number of DDDch (nDDDch)/animal/year for 360 farms housing weaned piglets. X-Axis: means of ATI and nDDDch/animal/year; Y-axis: differences between ATI and nDDDch/animal/year. Red lines: ±1.96 * standard deviation of the datasets calculated from the differences between ATI and nDDDch/animal/year.

**Figure 4:**
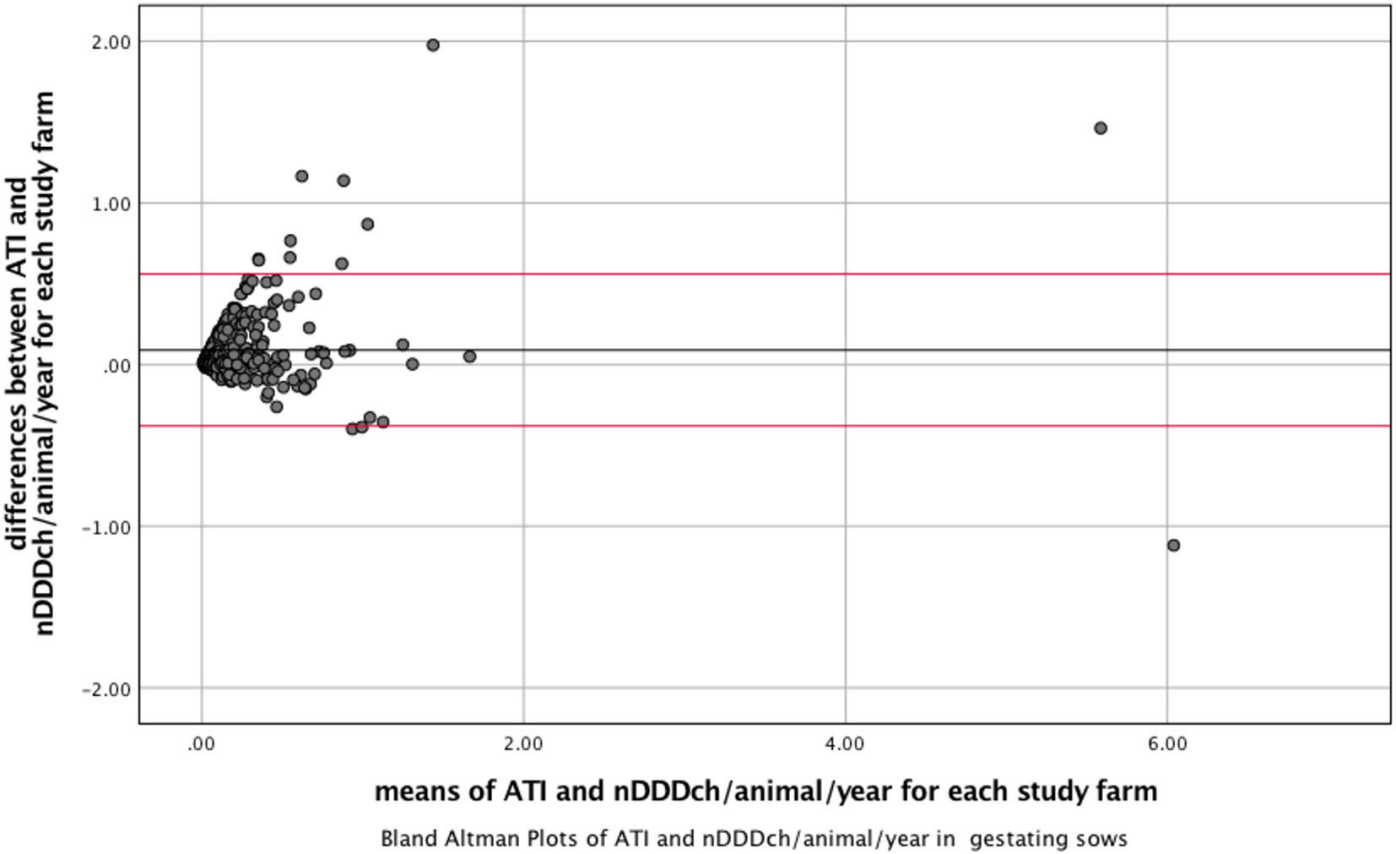
Bland-Altman Plots visualizing interrater reliability of the indicator Animal Treatment Index (ATI) and number of DDDch (nDDDch)/animal/year for 319 farms housing gestating sows. X-Axis: mean of ATI and nDDDch/animal/year; Y-axis: differences between ATI and nDDDch/animal/year. Red lines: ±1.96 * standard deviation of the datasets calculated from the differences between ATI and nDDDch/animal/year.

The correlation between both methods is further demonstrated with the data from weaned piglets as the age category with the best correlation between both indicators and with gestating sows, where the lowest correlation was found (Figures 1, 2). The interrater reliability is visualized by Bland-Altman plots for these age categories (Figures 3, 4). Bland Altman plots of weaned piglets showed five out of 360 (2%) to be outliers, while in gestating sows this was shown to be 13 out of 319 (4%).

## Discussion

In the present study we were able to assess the correlation of the indicators nDDDch/animal/year and ATI when measuring AMU on pig farms and the agreement in the definition of high usage farms. The number of treatment days NT and nDDDch calculated for the study farms showed marked differences for some active substances, while for others the results were similar. The degree of agreement between the two indicators was therefore most likely dependent on which antimicrobial substances were used in an age category.

The Spearman's Rho coefficients for correlations between the indicators ATI and nDDDch/animal/year observed were statistically determined as moderate (>0.6) for fattening pigs and gestating sows and as strong or very strong (>0.7) for suckling piglets, weaned piglets and lactating sows (37). The Kappa coefficient demonstrated a substantial agreement (>0.6) when defining 5% high usage farms for all age categories, except gestating sows. If defining 10 and 25% high usage farms, a moderate (>0.4) to substantial agreement was shown for all age categories except weaned piglets, where very good agreement (>0.8) could be shown (38).

The agreement of both indicators concerning the identification of high usage farms generally was the better, the smaller the percentage of farms was determined as high usage farms. If only 5% of the farms were determined as high users, even in age categories showing lower Kappa coefficient and lower correlations between both indicators, such as gestating sows or fattening pigs, more than 95% of the high usage farms were identified in agreement of both indicators. When 25% of the farms were determined as high users, the agreement between both indicators decreased to 75 and 77% in fattening pigs and gestating sows, respectively, due to the lower correlation and Kappa coefficient, while in weaned piglets with the best correlation and highest Kappa coefficient, the agreement still was 96%.

A final statement on whether the agreement between two indicators is sufficient or not cannot be made. Since only the degree of agreement can be shown, an extensive discussion is necessary to precisely evaluate which indicator is more suitable when planning a scientific study or when establishing and implementing a monitoring system for which a benchmark should be set (39).

Since the DDDch have been defined on the basis of SPCs in Switzerland, in order to achieve a good agreement between the indicators it is necessary that antimicrobial treatments are carried out as closely as possible to the recommendations found in the SPCs. Any change in dosage leads to a change in the number of DDD, but not in the number of treatments. According to our analyses, for some substances, significantly different levels of DDDch and ATI were calculated per age category. This result is probably due to the effect described above and is also responsible for both the outlier shown in our Bland-Altman Plots and decreased agreement when identifying high usage farms. On the other hand, the simultaneous calculation of both indicators may contribute to a mutual plausibility check of the results by, for example, identifying incorrect entries into the electronic treatment journal.

The divergent agreement between the indicators when comparing the different age categories could also be due to different indications, treatment patterns and antimicrobials used. For example, gestating sows often are suffering from lameness compared to weaned piglets which are more frequently affected by diarrhea. Further research is needed on this issue. Also, if actual weights at treatment deviate from the standard weights used for the calculation of DDDch, it may contribute to differences between DDDch and ATI. While, for example, a rather uniform weight during treatment can be expected for sows particularly in the case of suckling pigs, stronger deviations can be assumed between a newly born piglet with an average weight of 1.2 kg compared to a 4-week-old piglet with a multiple of this weight.

Differences between the indicators for AMU have been shown before (25, 34, 40). An increasing number of monitoring systems are being established worldwide, however, the harmonization of the measurement of usage has so far been less advanced (17). While in some countries the evaluation of antimicrobial usage is based on DDDs, an ATI or comparable methods are also used in other countries, including Switzerland (14, 15).

Treatment frequency calculations based on DDD are only an estimate of AMU on farms because they are calculated using standardized weights and doses. This indicator may be more appropriate if it is difficult to obtain precise information concerning the AMU, especially the number of treatments, the dosage used, and the weight of the treated animals. However, if this information can be collected reliably and accurately, the calculation of an ATI or treatment frequency based on used daily doses is feasible (27). On the other hand, it must also be taken into account that development of bacterial resistance also occurs outside the animal by excreted metabolites after antimicrobial therapy. In this situation, the quantities of antimicrobials used on a farm should also be taken into account (41, 42). These can be derived more reliably from DDDs, where standard weights and dosages are used and thus standardized antimicrobial quantities. To calculate the quantities of active substances used from an ATI, further information on the weight of the treated animals and the dosage applied is necessary.

In the present study it could be shown that despite the generally good agreement between the two indicators, a considerable proportion of the farms were nevertheless rated differently as high usage farms depending on the indicator used. In Switzerland, different monitoring systems are concurrently measuring AMU on farms. The use of different indicators may cause a considerable lack of compliance by farm managers when farm rating varies depending on the monitoring system used and may adversely affect the acceptance of such programmes.

Although many discussions are taking place regarding the comparability of different indicators, other factors should be taken into account which also influence the calculation of AMU: different data sources and deviating values provided, e.g., concerning the kept animals, may also result in different outcomes. In Switzerland, the numbers of animals reported to the veterinary authorities are in some cases significantly different from those used by the on-farm reproduction software, which is used as a data source for the number of animals on the farms (43).

Our earlier investigations have shown that within the Suissano/Safety+ Health Programme significant changes in the usage of antimicrobials could be achieved by only measuring and communicating levels of antimicrobial usage, without defining a benchmark (44). Thus, if a benchmark is not necessarily needed and the good correlation of both indicators found in the present study is taken into consideration, it may be of secondary importance which indicator to choose for the aim of reducing AMU.

It is also important to point out that irrespective of the method of calculation, a long-term reduction in the usage of antimicrobials while respecting animal health standards can only be feasibly achieved through close collaboration with veterinary professionals. Improving biosecurity as well as animal health e.g., by introducing vaccination protocols, has a positive impact on AMU (45). Ideally, any AMU monitoring programme will intensify veterinary advice and ensure a reduced AMU without impairing animal health rather than rating farms based only on their antimicrobial usage.

## Conclusions

In the present study, strong correlations and a broad agreement in identifying high usage farms could be demonstrated for the indicators nDDDch/animal/year and ATI. Nevertheless, depending on the indicator used a considerable proportion of the study farms were assessed differently. These differences varied by age category and were larger with a higher proportion of farms determined as high usage farms. These aspects have to be considered when designing scientific studies or monitoring systems and when deciding on which indicator to use.

## Data Availability Statement

The raw data supporting the conclusions of this article will be made available by the authors, without undue reservation.

## Author Contributions

DK drafted the manuscript. DK, CM, and XS designed and directed the study design. TE prepared data processing. All authors contributed to the article and approved the submitted version.

## Conflict of Interest

The authors declare that the research was conducted in the absence of any commercial or financial relationships that could be construed as a potential conflict of interest.

## Abbreviations

AMU: antimicrobial usage
AS: active substance
ATI: Animal Treatment Index
DDDch: Defined Daily Dose Switzerland
DCDch: Defined Course Dose Switzerland
DDDvet: Defined Daily Dose (EMA)
DCDvet: Defined Course Dose (EMA)
EMA: European Medicines Agency
Min: minimum
Max: maximum
NT: number of treatment days
nDDDch: number of DDDch
SD: standard deviation
SW: standard weight
VMP: Veterinary Medical Product.
